# Early-Onset Dengue Mimicking Neonatal Sepsis in a Term Neonate: A Case Report

**DOI:** 10.7759/cureus.107809

**Published:** 2026-04-27

**Authors:** Jwan Shekhy, Athl Mohammed, Hanan Alraeesi, Tarig Ahmed, Abeera Ummer

**Affiliations:** 1 Department of Pediatrics, Emirates Health Services / Khorfakkan Hospital, Sharjah, ARE; 2 Department of Pediatrics, Emirates Health Services / Khorfakkan Hospital, sharjah, ARE

**Keywords:** dengue related hospitalization, dengue thrombocytopenia, neonatal intensive care unit (nicu), neonatal sepsis mimic, newborn diseases, newborn rash, petechiae, vertical infectious disease transmission, vertical transmission

## Abstract

Neonatal dengue is an underrecognized infection that can mimic neonatal sepsis, leading to diagnostic challenges, particularly in endemic regions and in the context of perinatal or household exposure.

We report the case of a term male neonate, delivered by emergency cesarean section for abruptio placenta, who developed early respiratory distress, thrombocytopenia, and petechial rash shortly after birth. Initial management included respiratory support and empirical antibiotics for suspected sepsis; however, bacterial cultures remained negative. Dengue infection was confirmed by polymerase chain reaction and IgM serology in both the mother and the neonate. The infant improved with supportive care and had complete clinical and hematological recovery on follow-up.

This case highlights the importance of considering dengue in neonates presenting with sepsis-like features to enable timely diagnosis, avoid unnecessary antibiotic exposure, and ensure appropriate monitoring for potential complications.

## Introduction

Dengue is a globally significant mosquito-borne viral infection, with an estimated 100-400 million infections annually, predominantly in tropical and subtropical regions [1]. Although dengue primarily affects older children and adults, neonatal dengue is increasingly recognized, particularly in endemic areas. Vertical transmission, while considered uncommon, has been reported in up to 1-10% of dengue infections during pregnancy, particularly when maternal infection occurs in the late third trimester or peripartum period, likely through transplacental passage or exposure during delivery [2,3]. Despite this, perinatal dengue remains underdiagnosed.

Neonatal dengue typically presents within the first week of life with nonspecific features such as thrombocytopenia, petechiae, rash, and respiratory distress, which can closely mimic neonatal sepsis [4,5] and, less commonly, neurological involvement [6]. Given the overlap with bacterial sepsis and the potential for rapid clinical deterioration, maintaining a high index of suspicion is essential, especially in the context of maternal or household dengue infection. Distinguishing dengue from bacterial sepsis is clinically important, as management is primarily supportive and early recognition can help avoid unnecessary antibiotic exposure and invasive interventions.

## Case presentation

A male neonate, gestational age 39 weeks, birth weight 3.42 kg, was delivered on June 2024 by emergency cesarean section under general anesthesia due to abruptio placenta and decreased fetal heart sounds.

Perinatal history

A neonatal emergency response (Code Purple) was activated at the time of delivery, and the resuscitation team was present. The neonate was born flat, with meconium-stained liquor. Immediate resuscitation included drying and positive pressure ventilation with a T-piece. Within 1 minute, the baby cried with a heart rate of >100/min, good tone, and achieved pink color by 2 minutes. Apgar scores were 7, 9, and 9 at 1, 5, and 10 minutes, respectively. Initial tachypnea (RR 66-68/min) with nasal flaring prompted transfer to NICU at 15 minutes of life.

Initial NICU course (day 0)

The baby was started on CPAP (continuous positive airway pressure), PEEP (positive end-expiratory pressure) 7 cmH₂O, and FiO_2_ (fraction of inspired oxygen) 40% via RAM cannula. Cord blood gas showed metabolic acidosis (pH 7.18, HCO_3_ 14, base excess -13). At 3 hours of life, he became more stable on CPAP (PEEP 7, FiO_2_ 30%), had a respiratory rate of 50/min without retractions or grunting, and maintained saturation. He was hypoactive with fair sucking, and a petechial rash was noted over the right arm, chest, and neck. Sepsis work-up was collected, and empiric IV antibiotics were initiated.

Clinical progress

Days 1-2

**Figure 1:**
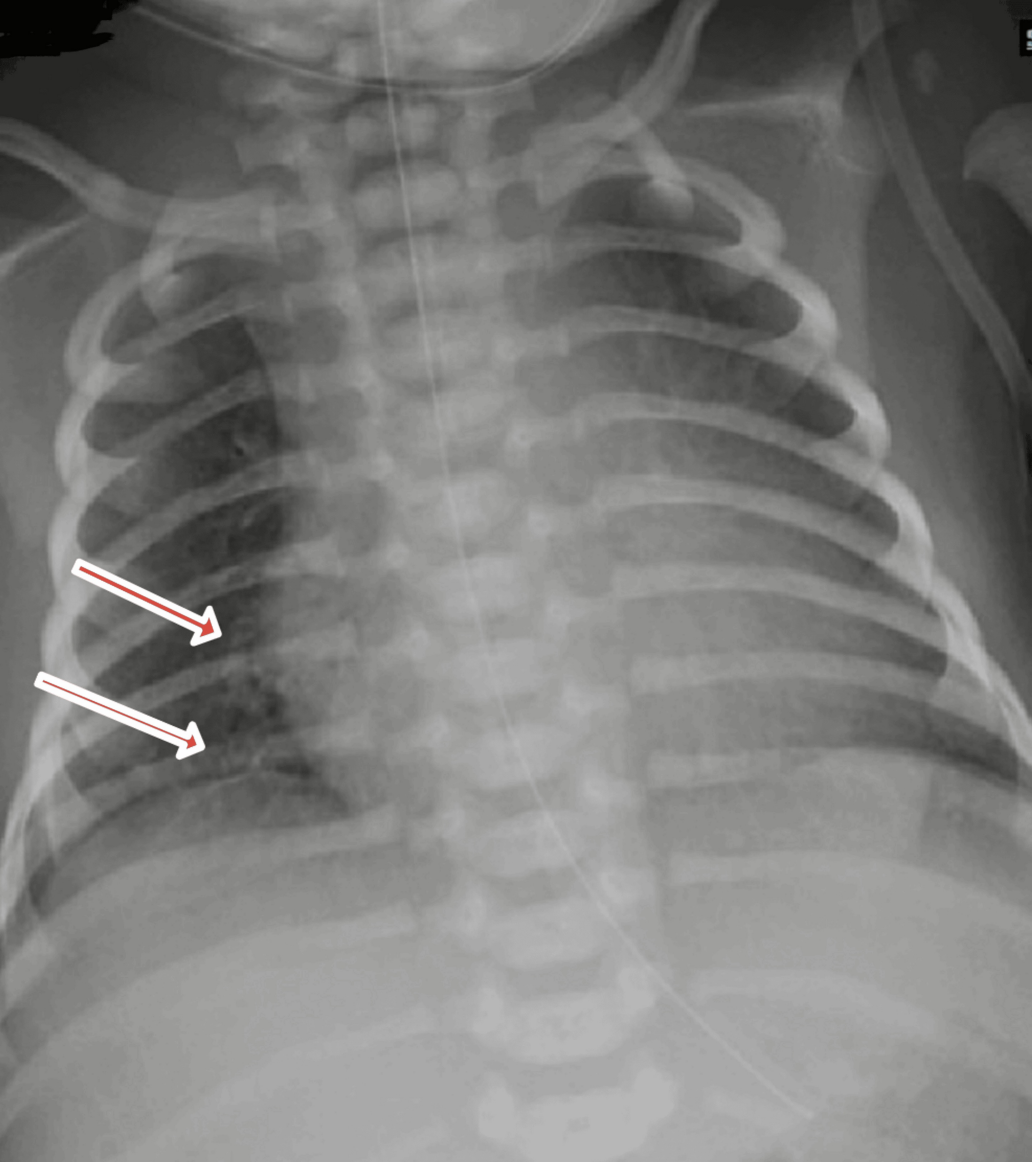
Chest X-ray on day 1 of life showing mild bronchovascular prominence (arrows)

The infant remained stable, active, pink, and without respiratory distress. A chest X-ray performed on day 1 of life showed mild bronchovascular prominence with otherwise normal findings, normal cardiac silhouette, and clear lung fields (Figure 1). He continued on non-invasive respiratory support. Enteral feeding was initiated via orogastric tube gradually. Urine and meconium output were adequate. Petechiae persisted on the right upper arm.

Day 3-4

The baby was successfully weaned to room air and remained stable with no respiratory distress. He tolerated direct breastfeeding, with good urine and stool output. Mild indirect hyperbilirubinemia was noted, with bilirubin levels consistently below phototherapy thresholds for age and gestation and therefore did not require phototherapy.

Serial hematological parameters showed leukocytosis with a downward trend and initial mild thrombocytopenia followed by recovery (Table 1).

**Table 1:** Hematological trends during hospital course (neonatal reference ranges) Values are interpreted using age-specific neonatal reference ranges, which differ from adult ranges, particularly for hemoglobin, hematocrit, and white blood cell counts in the early neonatal period. RDW, red cell distribution width

Parameter (Day of Life)	Day 1	Day 2	Day 3	Day 5	Reference Range
					(Term Neonate, Days 1–7 of Life)
WBC (×10³/µL)	63.13	41.79	25.13	19.8	9–30
Hemoglobin (g/dL)	16.1	15.8	15.6	14.7	14–22
Hematocrit (%)	49.1	47	45.5	45	44–65
Platelets (×10⁹/L)	101	63	98	186	150–450
RDW (%)	18.6	19.1	18.9	18.7	14–18
Neutrophils, absolute (×10³/µL)	25.78	21.19	14.34	9.99	3–14
Lymphocytes, absolute (×10³/µL)	25.16	13.2	6.72	5.56	3–9

Coagulation profile performed on day 3 of life was within normal limits (Table 2).

**Table 2:** Coagulation profile on day 3 of life PT, prothrombin time; INR, international normalized ratio; PTT, partial thromboplastin time

Parameter	Result	Reference Range (Term Neonate)
PT	9.9 sec	9–14
INR	0.85	0.8–1.2
PTT	40.8 sec	28–42

Cerebrospinal fluid analysis on day 2 of life showed markedly elevated protein and significant pleocytosis, both well above normal neonatal ranges; the sample was mildly traumatic. Culture and virology PCR was also sent (Table 3).

**Table 3:** Cerebrospinal fluid analysis (day 2 of life)

Parameter	Result	Reference Range (Term Neonate)
Appearance	Clear	Clear
Protein	1717 mg/dL	20–170 mg/dL
Glucose	2.5 mmol/L	2.2–3.9 mmol/L
WBC	148/µL	<20/µL
RBC	3,200/µL	<500/µL

Cerebrospinal fluid virology panel was negative for all tested pathogens, including bacterial and viral organisms commonly associated with neonatal meningitis (Table 4).

**Table 4:** Cerebrospinal fluid meningitis/encephalitis panel (day 2 of life)

Pathogen	Result	Interpretation
*Escherichia coli *K1	Not detected	Negative
Streptococcus agalactiae	Not detected	Negative
Streptococcus pneumoniae	Not detected	Negative
Haemophilus influenzae	Not detected	Negative
Listeria monocytogenes	Not detected	Negative
Neisseria meningitidis	Not detected	Negative
Cytomegalovirus (CMV)	Not detected	Negative
Enterovirus	Not detected	Negative
Herpes simplex virus 1 (HSV-1)	Not detected	Negative
Herpes simplex virus 2 (HSV-2)	Not detected	Negative
Human herpesvirus 6 (HHV-6)	Not detected	Negative
Human parechovirus	Not detected	Negative
Varicella-zoster virus (VZV)	Not detected	Negative
Cryptococcus neoformans/gattii	Not detected	Negative

C-reactive protein levels showed reassuring levels (Table 5).

**Table 5:** CRP trend during hospital course CRP, C-reactive protein

Parameter (Day of Life)	Day 1	Day 2	Day 3	Day 5	Reference Range
CRP (mg/L)	3.6	Not measured	1.7	1.3	<5

Management

The infant was commenced on empirical intravenous antibiotics at meningitic dose, including crystalline penicillin (50,000 units/kg/dose every 8 hours) and gentamicin (5 mg/kg once daily), and continued for a total duration of 5 days until cultures were confirmed negative along with supportive care. Respiratory support was initially provided with CPAP and was successfully weaned to room air by day 4 of life.

In view of thrombocytopenia and petechial rash, together with a history of dengue infection in a household contact (the elder sibling had laboratory-confirmed dengue infection two weeks prior to delivery, with a mild course and full recovery), congenital or perinatal dengue infection was suspected. The mother had no history of travel outside the UAE and remained well throughout pregnancy until developing an acute febrile illness on the day of delivery (peripartum period). Her initial laboratory evaluation, including platelet count, was within normal limits, with no features of severe dengue. Dengue-specific investigations (PCR and IgM serology) were performed simultaneously for both the mother and the neonate on day 2 of life (corresponding to day 2 of maternal illness), and were positive in both, confirming dengue infection (Table 6).

**Table 6:** Dengue-specific laboratory investigations

Test	Result	Reference Range / Interpretation
Dengue PCR	Detected	Not detected
Dengue IgM	Positive	Negative

Accordingly, dengue infection was confirmed. Vertical (congenital) transmission is considered likely but not definitive, supported by the early neonatal presentation and temporal association with maternal illness; however, postnatal acquisition cannot be entirely excluded. The infant was managed with supportive care, including monitoring of fluid balance, hemodynamic status, and hematological parameters. Blood culture collected on admission showed no growth at 48 hours and remained negative after 7 days of incubation, indicating no evidence of bacterial bloodstream infection. Cerebrospinal fluid culture showed no growth on day 3 of life, effectively excluding bacterial meningitis.

By the end of the first week of life (day 7 of life), the infant was active, maintaining normal vital signs, and in room air. The petechial rash had nearly resolved, and platelet counts showed a consistent upward trend toward normalization. There were no bleeding manifestations or features of severe dengue. The infant was discharged home in stable condition with instructions for close follow-up.

At three days post-discharge, the infant was reviewed in the outpatient clinic and was well, active, feeding adequately, with normal vital signs, no recurrence of rash, and stable hematological parameters.

At two weeks post-discharge, follow-up assessment showed continued normal growth and activity, complete resolution of thrombocytopenia, no clinical concerns, and overall normal examination findings.

## Discussion

Neonatal dengue remains an underrecognized clinical entity due to its nonspecific presentation and significant overlap with neonatal sepsis, particularly in endemic regions [7,8]. This overlap often leads to diagnostic uncertainty and the initiation of empirical antibiotic therapy before a viral etiology is considered. This case highlights the importance of maintaining a high index of suspicion for dengue infection in neonates presenting with thrombocytopenia, respiratory distress, and rash, especially in the presence of maternal or household exposure. In our case, the history of recent dengue infection in both the mother and sibling strongly supports the likelihood of vertical or early postnatal transmission, both of which have been increasingly reported in the literature [2,9].

Vertical and perinatal transmission of dengue, although uncommon, is now well documented, particularly when maternal infection occurs in the late third trimester or near the time of delivery [2,10]. The pathophysiology is thought to involve transplacental passage of the virus or exposure during the peripartum period. Neonates may present within the first week of life with clinical features such as thrombocytopenia, petechiae, fever, and respiratory distress, which can closely resemble neonatal sepsis or other infectious conditions [7,10]. This overlap contributes to underdiagnosis and may delay appropriate recognition of dengue infection.

The diagnostic challenge is further compounded by laboratory findings that may mimic bacterial or viral central nervous system infections. In this case, CSF pleocytosis and elevated protein initially raised suspicion for meningitis. However, sterile cultures and a negative meningitis/encephalitis PCR panel supported a non-bacterial etiology, a phenomenon previously described in neonatal dengue cases [11]. Definitive diagnosis relies on virological confirmation, with dengue PCR and IgM serology playing a crucial role in early and accurate identification of infection [12].

Thrombocytopenia and petechiae are among the most consistent and important clinical clues in neonatal dengue and should prompt targeted evaluation, particularly in endemic settings [4]. Although neurological complications such as encephalopathy and seizures have been reported in perinatal dengue, they were not observed in this case, indicating a relatively mild clinical course [6]. Close monitoring remains essential, as disease progression can be unpredictable.

Management of neonatal dengue is primarily supportive, focusing on careful monitoring of fluid balance, hemodynamic status, and hematological parameters to prevent complications such as bleeding, plasma leakage, and shock [12]. Judicious fluid management is particularly important to avoid both hypovolemia and fluid overload. In this case, early recognition and appropriate supportive care resulted in a favorable outcome and prevented unnecessary escalation of antibiotics and invasive interventions.

Overall, this case reinforces the need for increased awareness among neonatologists and pediatricians regarding neonatal dengue as a differential diagnosis in sepsis-like presentations. Early consideration, appropriate diagnostic testing, and supportive management are key to improving outcomes and avoiding unnecessary treatments.

This report is subject to certain limitations that should be acknowledged. Although maternal and neonatal dengue infection were confirmed (PCR and IgM obtained on day 2 of life), vertical transmission cannot be definitively established. The mother developed fever on the day of delivery, and a household contact (sibling) had confirmed dengue infection two weeks prior; however, the neonate had no direct postnatal contact with either the mother or sibling and was admitted to the NICU immediately after birth, which reduced the likelihood of horizontal transmission. Additionally, cerebrospinal fluid interpretation was limited by a traumatic sample, which may have contributed to elevated protein and cell counts. Finally, as a single case report, the findings are not generalizable and should be interpreted with caution.

## Conclusions

Neonatal dengue should be considered in neonates presenting early with thrombocytopenia, petechiae, and sepsis-like features, particularly with maternal or household exposure. Early dengue testing alongside sepsis work-up helps avoid unnecessary antibiotic use. Most cases follow a self-limited course with supportive care, but close monitoring for bleeding and hemodynamic instability is essential.
